# Emergency Department Presentations During Dry and Humid Heatwaves: A Case‐Crossover Study in the Northern Territory, Australia

**DOI:** 10.1029/2025GH001562

**Published:** 2026-05-29

**Authors:** Rowena Boyd, Alyson Wright, Nicolas Borchers‐Arriagada, Paul Fox‐Hughes, Fay H. Johnston, Paul Burgess, Tracy Ward, Sharon L. Campbell

**Affiliations:** ^1^ Department of Health Public Health Directorate Darwin NT Australia; ^2^ Menzies Institute for Medical Research University of Tasmania Hobart TAS Australia; ^3^ Bureau of Meteorology Hobart TAS Australia; ^4^ Department of Health Public Health Services Hobart TAS Australia

**Keywords:** heatwave, humidity, health, tropical, arid

## Abstract

In a rapidly warming climate, heatwaves pose an increasing threat to human health. However, there is limited knowledge of heatwave impacts on health outcomes, or the role of humidity in the tropical and arid climates of Australia's Northern Territory (NT). Using a space‐time‐stratified case‐crossover design and conditional Poisson regression models, we analysed the association between heatwaves and emergency department (ED) presentations from 2001 to 2023, across all six NT public hospitals. Heatwaves were identified using the Excess Heat Factor (EHF) method, with both temperature‐only (heatwaves_T) and temperature‐plus‐humidity, heat‐index (heatwaves_TH) metrics. We undertook sub‐group analyses by sociodemographic characteristics and principal diagnosis. All‐cause ED presentations increased by 4.4% (RR = 1.044, 95%CI 1.018–1.071) for severe/extreme and 1.6% (RR = 1.016, 95%CI 1.002–1.030) for low‐intensity heatwaves_T. For heatwaves_TH, presentations increased by 6.1% (RR = 1.061, 95%CI 1.025–1.098, severe/extreme) and 0.9% (RR = 1.009, 95%CI 0.995–1.024, low‐intensity). Subpopulation increases for severe/extreme heatwaves_T occurred for ages 19–49 years (RR = 1.052; 95%CI 1.018–1.087), visitors (RR = 1.162, 95%CI 1.038–1.301) and skin conditions (RR = 1.116, 95%CI 1.048–1.189). Specific to severe/extreme heatwaves_TH, presentations increased for Aboriginal peoples (RR = 1.059, 95%CI 1.006–1.114), ages 50–64 years (RR = 1.141, 95%CI 1.059–1.230) and cardiovascular conditions (RR = 1.111, 95%CI 1.015–1.216). Comparing heatwave indexes, 57.1% of heatwave_T days were not captured by heatwave_TH, and conversely 49.6% of heatwaves_TH days were not captured by heatwave_T. These findings call for dual heatwave warning systems in the NT, incorporating both EHF temperature and heat‐index, and further humidity‐inclusive studies in varied climates. Preventative interventions should target high‐risk populations, prioritizing resources for severe and extreme heatwaves.

## Plain Language Summary

Our research investigates how dry‐heat and humid heatwaves impact emergency department presentations in the Northern Territory, Australia. We find that both dry‐heat and humid heatwaves cause emergency department presentations to rise, particularly for visitors during dry‐heat heatwaves and for Aboriginal people during humid heatwaves. This means that heatwave warning systems must also include measures for humidity, and target high‐risk groups.

## Key Points

- The Northern Territory's (NT) annual number of heatwave days is rising, posing a growing threat to human health
- Both dry‐heat and humid‐heat heatwaves increase emergency department presentations across the NT's tropical and arid climates
- Heatwave warnings must include humidity measures to guide population health promotion and plan for increased emergency department demand

## 1 Introduction

Climate change is driving more frequent and intense heatwaves globally, including in Australia where average temperatures have risen by 1.51°C since 1910 (Bureau of Meteorology & CSIRO, 2024). Heatwaves have killed more people in Australia than all other natural disasters combined (Coates et al., 2014), and are known to impact health through increased mortality, ambulance retrievals, emergency health presentations and hospital admissions (Campbell et al., 2018). Extreme heat can overwhelm the body's thermoregulatory capacity, triggering detrimental health pathways that lead to ischemia, heat cytotoxicity, inflammation, disseminated intravascular coagulation, or rhabdomyolysis which can critically damage the brain, heart, intestines, kidneys, liver, lungs, and pancreas (Mora et al., 2017). The greatest impact is felt by populations with the most limited thermoregulatory and resource capacity, such as infants and the elderly, those with underlying health conditions or people living in poor quality housing (Mason et al., 2022; Mora et al., 2017). Minimizing heat‐related health impacts depends on global reductions in greenhouse gas emissions, local preventative measures such as increasing shaded areas, heatwave warning systems, public education, and risk communication incorporating Indigenous knowledge, particularly targeted toward at‐risk populations (Australian Government, 2023).

Despite these global findings and recognition that extreme heat will increase in the Northern Territory (NT), there is a lack of studies within tropical and arid climates, rural, remote and Indigenous populations (Bhatta et al., 2023; Northern Territory Government, 2020). Consequently, the heat‐related morbidity burden in the NT is largely unknown. A recent NT mortality study showed increased risk of heat‐related deaths in 2000–2019, with similar mortality risk for Aboriginal and non‐Aboriginal populations (Quilty et al., 2023). Quilty et al. (2023) discussed the comparative adaptive strengths of non‐Aboriginal access to modern infrastructure and technology and Aboriginal sociocultural adaptations to extreme heat based on generational learnings.

### 1.1 Northern Territory Setting

**Figure 1:**
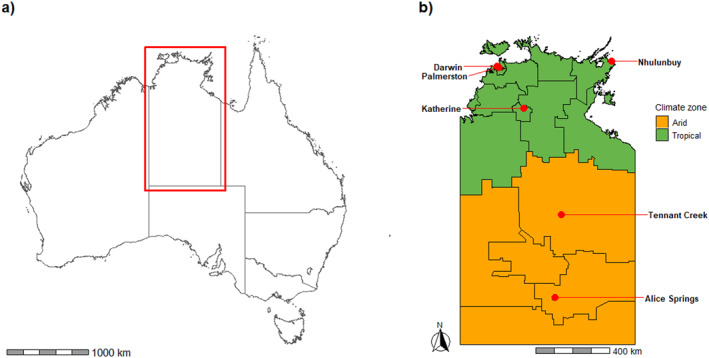
(a) Map of Australia, Northern Territory identified by red box; (b) Map of Northern Territory by climatic zone, Statistical Area 2 (SA2) regions outlined by black lines and location of public hospitals (red dots).

The NT climate is one of extremes. The monsoonal, tropical zone covers the top third of the NT, while the bottom two‐thirds is known as the arid zone (Figure 1). The tropical zone experiences high maximum temperatures averaging over 30° Celsius throughout the year, with a distinct wet season from November to March characterized by high humidity and minimum temperatures, with 95% of annual rainfall. In contrast, during the dry season (April–October), the tropical zone experiences lower humidity and minimum temperatures, with very little rainfall. The southern arid zone has four distinct seasons, a generally hot summer (December–February), cool winter (June–August) and intermediate transition seasons of spring (September–November) and autumn (March–May). The NT consists of some of the most remote areas in Australia, as classified by the Australian Bureau of Statistics (ABS), and is sparsely populated with only 232,600 residents, approximately 80% of whom live in the tropical zone (Northern Territory Government, 2022). Aboriginal and Torres Strait Islander peoples (henceforth respectfully referred to as Aboriginal peoples) account for 26% of the population, proportionately the highest of all jurisdictions in Australia (Australian Bureau Of Statistics, 2021). There are six public hospitals with emergency departments (ED), while the only private hospital does not have an accompanying ED.

### 1.2 Excess Heat Factor (EHF) and Humidity

The temperature derived “Excess Heat Factor” (EHF) is an index developed and utilized by the Australian Bureau of Meteorology (BoM) to identify and define heatwave events, forming the thresholds for Australia's heatwave warning system (Bureau of Meteorology, 2025). The BoM further classifies heatwaves severity into low‐intensity, severe, or extreme based on how strongly the event's heat stress exceeds what is normally experienced at that location. This index accounts for acclimatization by defining heatwaves as three or more consecutive days of unusually high (minimum and maximum) temperatures relative to the past 30 days. While the EHF based on temperature values alone has been shown to correlate well with measurable heat impacts on human health, there is limited evidence on the influence of higher humidity on health (Bureau of Meteorology, 2024a; Oppermann et al., 2017; Scalley et al., 2015). Noting the absence of heatwave impact studies on human health in Australia's tropical regions, modeling undertaken by Nairn et al. (2022) showed that the application of EHF in tropical regions of Darwin (NT) and Port Hedland (Western Australia) failed to capture instances of extreme humidity in the absence of elevated temperature. Nairn et al. (2022) found employing the EHF calculation with incorporation of relative humidity as well as temperature as a heat index can overlook extreme heat periods characterized by exceptionally hot and dry conditions, leading to recommendations that epidemiological studies in tropical regions apply both indexes (EHF temperature‐only and EHF temperature‐plus‐humidity) (Nairn et al., 2022).

### 1.3 Study Aim

We aimed to (a) analyze the association between heatwaves and ED presentations in the NT, (b) examine whether results are sensitive to EHF indexes with and without humidity, (c) identify variations across sub‐populations and disease categories, and (d) identify variation across climate zones.

Our study addresses gaps in knowledge of the health impacts of heatwaves in tropical, arid and remote regions as measured by presentations to NT EDs during heatwave events. We add to the limited evidence of heatwave impacts on Aboriginal peoples. Further, we improve knowledge of the suitability of a temperature‐only derived heatwave warning system for tropical and arid climates, assessing whether incorporation of humidity reveals health impacts not captured by the current temperature‐only EHF index.

## 2 Methods and Data

### 2.1 Study Design

We used a space‐time‐stratified case‐crossover study, which is commonly applied in environmental epidemiology where the study population is exposed to a short‐term event (Wu & Guo, 2021). This approach allows for the use of location‐specific time series data, where aggregated counts are used instead of individual data. For this design, a stratum, a categorical variable grouping a location‐specific day of the week, within the same month and same year, is defined. This method yields similar results to those of applying a conditional logistic regression on individually matched case‐control data. Similarly, this method, by design, allows for control of time‐invariant, potentially confounding factors such as seasonality, day‐of‐the‐week effects, long‐term trends, and spatial variation (Wu & Guo, 2021).

### 2.2 Exposure Data

Heatwaves were identified by extracting: (a) daily mean temperature data from BoM 5 km^2^ gridded climatology data (Bureau of Meteorology, 2024b) utilizing the “exactextractr” R package (Baston, 2024); and (b) relative humidity data derived from meteorological data from the fifth generation European Centre for Medium‐Range Weather Forecasts (ECMWF) ERA5‐Land reanalysis data set (Muñoz Sabater, 2019). ERA5‐Land hourly 9 km^2^ gridded temperature and dew point data were used to derive daily means and relative humidity, using the R “weathermetrics” package (Anderson et al., 2013, 2016). These data were mapped to the 68 Statistical Area 2 (SA2) regions in which the NT is divided, as defined by ABS 2016 boundaries (Australian Bureau Of Statistics, 2023). SA2s are medium‐sized general‐purpose areas with a mean population of 3,385 people and designed to represent communities with shared social and economic interactions. Exposure to a heatwave was defined as living within an SA2 region at the time of the event. Heatwave “days” refers to location‐days, with each of 68 SA2 regions contributing to 365 days per year (366 days in leap years).

EHF and low‐intensity, severe and extreme heatwave intensities were calculated in accordance with Nairn and Fawcett (2015) which are well described by BoM (2025, pages 16–17). Our reference period for the 85th percentile severity thresholds were 1960–2011 due to data availability. Two separate EHF indexes were derived as described in Nairn et al. (2022):

- EHF temperature index: hereafter “heatwave(s)_T” utilizing temperature data alone
- EHF heat index: hereafter “heatwave(s)_TH” utilizing temperature and relative humidity data

As severe and extreme heatwaves were relatively rare, they were combined for analysis. We further aggregated SA2s to the larger climatic zones of hot, humid summer (tropical region) or hot, dry summer (arid region), as defined in Figure 1 (Bureau of Meteorology, 2024a). These zones were analyzed separately given their differing meteorological characteristics.

### 2.3 Outcome Data

ED data from 1 January 2001 to 31 December 2023 for all six public hospitals were extracted from the NT Department of Health data warehouse. Locations of the major centers hosting NT public hospitals are shown in Figure 1. Data extracts included date, time and hospital of presentation, demographic characteristics (gender, age and Indigenous status) collected at the point of care, SA2 of residential address and primary diagnosis in accordance with the emergency care International Classification of Disease (ICD‐10‐AM) diagnostic shortlist (Independent Health and Aged Care Pricing Authority, 2024). We used binary sex categorization (male/female) as reported in the individual's health record.

Socioeconomic disadvantage was classified using 2016 ABS index of relative socio‐economic disadvantage (IRSD) deciles which were mapped to SA2 residential address and categorized as most disadvantaged (IRSD decile 1‐3), moderate (IRSD decile 4‐7), and most advantaged (IRSD decile 8‐10) (Australian Bureau Of Statistics, 2018).

Residential address was used to determine status as either residential (NT address) or visitor (non‐NT address). Place of exposure for visitors and unknown or missing addresses were mapped to the corresponding SA2 of the hospital they attended.

### 2.4 Covariate Data

Modeled data for particulate matter with a diameter less than 2.5 µm (PM_2.5_) were obtained from the Clean Air and health Research Data and Analysis Technology (CARDAT) platform (Hanigan et al., 2023, 2025) to adjust for the influence of air pollution on health service utilization. We conducted a sensitivity analysis, excluding PM_2.5_ given debate about whether air quality functions as a mediator or confounder in the heat‐health relationship (Buckley et al., 2014). NT and regional public holidays were sourced from the NT Office of the Commissioner for Public Employment.

### 2.5 Analysis

Conditional Poisson (quasi‐Poisson) regression models were used to analyze the association between heatwave exposure and ED presentations, reporting risk ratios (RR) and 95% confidence intervals (95%CI). Models were applied for:

- the entire population, inclusive of all‐cause ED presentations,
- sub‐population analyses for demographic characteristics including Indigenous status, residential status, sex, age group and IRSD,
- sub‐population analysis by climatic zone inclusive of tropical or arid climate, and
- the most common diagnostic reasons for ED presentations, diabetes and the effects of heat and light.

All models were controlled for PM_2.5_ and public holidays. Analyses were undertaken in R version 4.4.2 utilizing the gnm() package (Campbell et al., 2024; Turner et al., 2023; Wu & Guo, 2021). The equation used for each model was:

$$\text{gnm}\left(\text{Number}\_\text{of}\_\text{Cases}\;∼\;\text{HeatwaveSeverity}+\text{PublicHoliday}+{\text{PM}}_{2.5},\text{data}=\text{dataframe},\text{family}=\text{quasipoisson},\text{eliminate}=\text{factor}\left(\text{stratum}\right)\right.)$$

where the stratum is a concatenated variable of SA2:Year:Month:Day of the week (e.g., DarwinAirport:2001:1:5).

Ethics approval was obtained from the Human Research Ethics Committee of the Northern Territory Department of Health and Menzies School of Health Research, Reference 2024–4884.

## 3 Results

### 3.1 Descriptive Summary of Emergency Department (ED) Presentations

Between 1 January 2001 and 31 December 2023, there were 3,191,418 presentations to EDs in the NT. There were 184,126 presentations on heatwave_T days, and 152,252 on heatwave_TH days (Table 1). Aboriginal peoples comprised 48.3% (*n* = 88,928) and 49.2% (*n* = 74,968) of the respective heatwave cohorts, and visitors to the NT accounted for a minority of presentations at 5.4% (*n* = 9,923) and 5.7% (*n* = 8,662) respectively. The arid climate region had a lower proportion of presentations (*n* = 54,088, 29.4%) for heatwaves_T, however slightly higher representation for heatwaves_TH (*n* = 54,868, 36.0%). Over half of presentations were in 19–49 year olds, with 51.5% (*n* = 94,768) for heatwaves_T, and 52.5% (*n* = 79,527) for heatwaves_TH.

**Table 1:** Characteristics and Number of Cases Presenting to an Emergency Department on Heatwave Location‐Days^a^ by Intensity, Temperature Only and Temperature‐Plus‐Humidity Heatwaves, Northern Territory, 2001–2023

	Heatwave—Temperature only		Heatwave—Temperature + humidity	
Total number of cases	184,126		152,252	
	Low intensity	Severe/extreme	Low intensity	Severe/extreme
Characteristic	Number of cases (percentage of total)		Number of cases (percentage of total)	
Total	151,129	32,997	131,892	20,360
Indigenous status				
Aboriginal	72,931 (48.3%)	15,997 (48.5%)	65,751 (49.9%)	9,217 (45.3%)
Non‐Aboriginal	78,006 (51.6%)	16,952 (51.4%)	66,023 (50.1%)	11,117 (54.6%)
Unknown	192 (0.1%)	48 (0.1%)	118 (0.1%)	26 (0.1%)
Residential status				
Resident	142,272 (94.1%)	30,983 (93.9%)	123,749 (93.8%)	19,105 (93.8%)
Visitor	8,094 (5.4%)	1,829 (5.5%)	7,550 (5.7%)	1,112 (5.5%)
Unknown	763 (0.5%)	185 (0.6%)	593 (0.4%)	143 (0.7%)
Sex				
Male	76,216 (50.4%)	16,540 (50.1%)	66,194 (50.2%)	10,363 (50.9%)
Female	74,836 (49.5%)	16,436 (49.8%)	65,642 (49.8%)	9,983 (49.0%)
Unknown	77 (0.1%)	21 (0.1%)	56 (0.0%)	14 (0.1%)
Climate zone				
Tropical	105,761 (70.0%)	24,277 (73.6%)	82,359 (62.4%)	15,025 (73.8%)
Arid	45,368 (30.0%)	8,720 (26.4%)	49,533 (37.6%)	5,335 (26.2%)
Index of Relative Socio‐economic Disadvantage (IRSD)				
Least advantage (1–3)	56,530 (37.4%)	12,367 (37.5%)	51,009 (38.7%)	6,710 (33.0%)
Moderate advantage (4–6)	49,220 (32.6%)	11,075 (33.6%)	43,128 (32.7%)	6,139 (30.2%)
Most advantage (7–10)	43,761 (29.0%)	9,169 (27.8%)	36,443 (27.6%)	7,272 (35.7%)
Unknown	1,618 (1.1%)	386 (1.2%)	1,312 (1.0%)	239 (0.0%)
Age group				
<5 years	16,644 (11.0%)	3,441 (10.4%)	14,452 (11.0%)	2,151 (10.6%)
5–18 years	20,181 (13.4%)	4,350 (13.2%)	17,004 (12.9%)	2,655 (13.0%)
19–49 years	77,880 (51.5%)	16,888 (51.2%)	69,071 (52.4%)	10,456 (51.4%)
50–64 years	24,302 (16.1%)	5,490 (16.6%)	20,947 (15.9%)	3,350 (16.5%)
≥65 years	12,028 (8.0%)	2,805 (8.5%)	10,298 (7.8%)	1,730 (8.5%)
Unknown	94 (0.1%)	23 (0.1%)	120 (0.1%)	18 (0.1%)
Medical condition				
Infectious (A00‐A99, B00‐B99, U07.1‐U07.2)	7,525 (5.0%)	1,689 (5.1%)	6,710 (5.1%)	972 (4.8%)
Diabetes (E10‐E11, E14)	238 (0.2%)	60 (0.2%)	201 (0.2%)	31 (0.2%)
Mental/behavioral (F00‐F99, R44, R45)	6,558 (4.3%)	1,402 (4.2%)	5,899 (4.5%)	818 (4.0%)
Cardiovascular (I00‐I51, R00.1‐R00.2, R07.1, R07.4)	7,877 (5.2%)	1,778 (5.4%)	6,748 (5.1%)	1,079 (5.3%)
Respiratory (J00‐J99, R04.0, R04.2, R05, R06.0‐R06.2, R06.4, R06.8, R07.0, R09.1, R09.2, R09.89)	14,256 (9.4%)	3,093 (9.4%)	12,249 (9.3%)	1,819 (8.9%)
Digestive (K00‐K92, R10, R11, R13, R17, R18, R19.0, R19.89)	12,988 (8.6%)	2,934 (8.9%)	11,343 (8.6%)	1,697 (8.3%)
Skin (L00‐L98, R20.8, R21, R22.9, R23.8)	10,051 (6.7%)	2,182 (6.6%)	9,071 (6.9%)	1,354 (6.7%)
Musculoskeletal (M00‐M95, R25.2, R26.8, R29.89)	9,613 (6.4%)	2,150 (6.5%)	8,176 (6.2%)	1,240 (6.1%)
Urinary (N00‐N39)	3,668 (2.4%)	815 (2.5%)	3,187 (2.4%)	509 (2.5%)
Injury (S00‐S99, T00‐T66, T68‐T88)	30,810 (20.4%)	6,717 (20.4%)	26,024 (19.7%)	4,124 (20.3%)
Effects heat and light (T67^b^)	154 (0.1%)	47 (0.1%)	125 (0.1%)	18 (0.1%)

^a^ Location‐days refers to the total number of daily observations contributed by each region, that is, 365 days per year (or 366 in leap years) multiplied by 68 Statistical Area Level 2 (SA2) regions.

^b^ X30 not included in ICD‐10‐AM emergency care diagnostic shortlist.

Injury, inclusive of trauma‐inducing physical, chemical, electrical, thermal (burns, hypothermia), water immersive, allergy, or toxin exposures affecting internal organs and external structures, was the most common reason for presentation, accounting for 20.4% (*n* = 37,527) of presentations in heatwaves_T and 19.8% (*n* = 30,148) in heatwaves_TH. Respiratory illness was the second most common, contributing to 9.4% of ED presentations (*n* = 17,349) for heatwaves_T and 9.2% (*n* = 14,068) for heatwaves_TH. Presentations recorded as 'effects of heat and light' (ICD‐10 code T67) were rare, with 201 cases (0.1%) for heatwaves_T and 143 cases (0.1%) for heatwaves_TH.

### 3.2 Temperature, Humidity and Heatwave Frequency and Trends

Table 2 shows mean temperature and humidity for five SA2's representative of the main population centers in the NT. Mean temperature was highest in tropical Darwin (28.3°C, range 19.1°C–32.9°C) while arid Alice Springs recorded the lowest mean temperature and much wider range (21.1°C, 5.3°C–36.8°C). Nhulunbuy in the tropical north‐east of the NT had the highest mean relative humidity (75.9%) and the most heatwave days for both heatwave indexes.

**Table 2:** Temperature and Relative Humidity Mean and Range for Selected Statistical Area 2 (SA2) Representing Major Northern Territory Population Centres, and Number of Heatwave Days by Severity and Excess Heat Factor Index, 2001–2023

SA2	Temp. Mean (range) °C	Relative humidity mean (range) %	Number of heatwave days							
			Temperature only				Temperature + humidity			
			Low intensity	Severe intensity	Extreme intensity	Total heatwaves	Low intensity	Severe intensity	Extreme intensity	Total heatwaves
Darwin City	28.3 (19.1–32.9)	66.5 (17.7–95.8)	305	72	2	379	287	51	9	347
Nhulunbuy	27.2 (19.6–33.8)	75.9 (48.9–94.7)	523	90	12	625	404	79	20	503
Katherine	27.2 (14.4–35.9)	51.5 (10.5–98.0)	455	123	4	582	268	26	1	295
Tennant Creek	26.0 (8.6–38.1)	33.9 (4.2–96.6)	305	69	4	378	242	21	1	264
Alice Springs^a^	21.1 (5.3–36.8)	34.4 (5.4–95.4)	320	56	1	377	367	39	3	409

^a^ SA2 name: Charles. 2001‒2005 to the end period of 2019‒2023, the percentage of days meeting heatwave conditions rose by 1.7 percentage points to 4.9% for low‒intensity heatwaves in tropical conditions, by 2.5 percentage points to 5.0% in arid conditions (heatwaves_T / heatwaves_TH). Severe/extreme heatwaves_T increased by 0.6 percentage points to 1.5% for tropical conditions, however there were marginal changes for heatwaves_TH (Table S1 in Supporting Information S1).

Across 23 years and 68 NT SA2 regions (571,200 location‐days), 5.4% (30,767) of location‐days met heatwave_T conditions and 4.6% (26,183) met heatwave_TH conditions. The majority of heatwaves were of low‐intensity with fewer days meeting the severe or extreme intensity criteria. Severe heatwaves occurred on 5,454 days (17.7%) for heatwaves_T and 3,256 days (12.4%) for heatwaves_TH. Extreme intensity days were rare but were more common for heatwave_TH (572 days, 2.2%) than the heatwave_T days (200 days, 0.7%). Heatwaves primarily occurred between October and March regardless of heatwave index of heatwave_T (30,767 days, 98.0%) or heatwave_TH (25,523 days, 97.5%).

**Figure 2:**
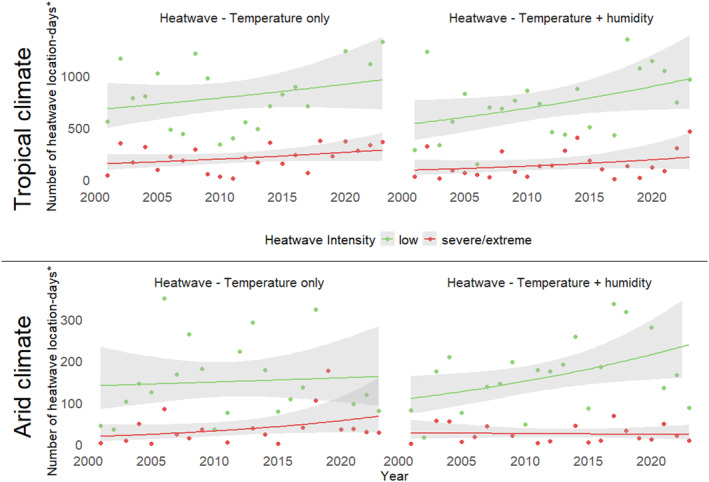
Number of heatwave location‐days* and trendlines† by tropical and arid climate, by heatwave index (temperature‐only and temperature‐plus‐humidity), Northern Territory, 2001–2023. * Location‐days refers to the total number of daily observations contributed by each region, that is, 365 days per year (or 366 in leap years) multiplied by 68 Statistical Area Level 2 (SA2) regions. †Trend‐lines are derived from generalized linear models (Gamma family with a log link) with the gray ribbon representing the 95% confidence interval.

From 2001 to 2023, there was an increasing number of heatwave days observed in both tropical and arid regions for both heatwaves_T and heatwaves_TH (Figure 2). Comparing the initial study period of 2001–2005 to the end period of 2019–2023, the percentage of days meeting heatwave conditions rose by 1.7% points to 4.9% for low‐intensity heatwaves in tropical conditions, by 2.5% points to 5.0% in arid conditions (heatwaves_T/heatwaves_TH). Severe/extreme heatwaves_T increased by 0.6% points to 1.5% for tropical conditions, however there were marginal changes for heatwaves_TH (Table S1 in Supporting Information S1).

Comparison between heatwave indexes identified 57.1%, (17,580 days) extremely hot, “dry” days identified by the heatwaves_T index were classified as a non‐heatwave day by the heatwaves_TH index (Table 3). Conversely, 49.6% (12,996 days) very humid heatwave days identified by the heatwaves_TH index were not identified as a heatwave day by the heatwaves_T index.

**Table 3:** Comparison of Number of Heatwave Location‐Days^a^ Identified by Heatwave Index, Northern Territory, 2001–2023

			Heatwave ‐ Temperature only	
			(Heatwave_T)	
			30,767 heatwave days	
			Non‐heatwave days	Heatwave days
Heatwave—Temperature + humidity (Heatwave_TH)	26,183 heatwave days	Non‐heatwave days	527,437	17,580 (57.1%^b^)
		Heatwave days	12,996 (49.6%^c^)	13,187

^a^ Location‐days refers to the total number of daily observations contributed by each region, that is, 365 days per year (or 366 in leap years) multiplied by 68 Statistical Area Level 2 (SA2) regions.

^b^ 57.1% is calculated from the column total 17,580/30,767.

^c^ 49.6% is calculated from the row total 12,996/26,183.

### 3.3 Association Between Heatwaves and ED Presentations

**Figure 3:**
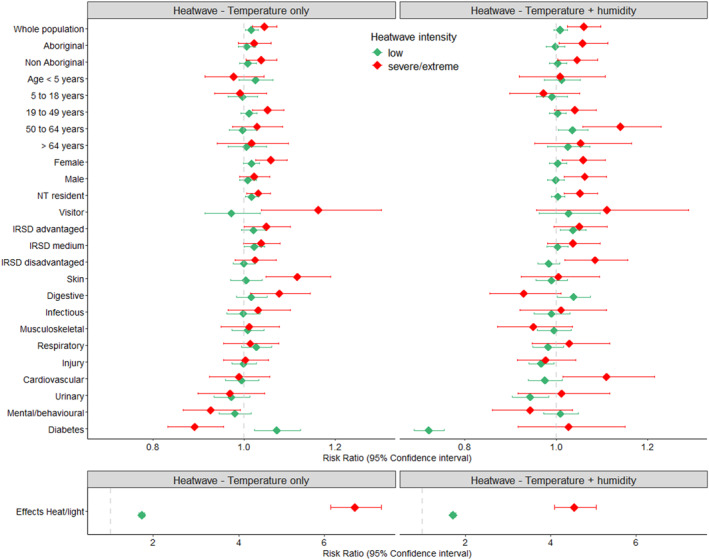
Association between NT emergency department presentations and heatwaves (temperature‐only and temperature‐plus‐humidity), by heatwave index, sociodemographic characteristic, presenting diagnosis and heatwave intensity, 2001–2023. Effects of light and heat on a separate chart due to substantial differences in scale.

All‐cause ED presentations had a significant increase of 4.4% (RR 1.044, 95% CI 1.018–1.071) for severe/extreme intensity and 1.6% (RR 1.016, 95% CI 1.002–1.030) for low‐intensity heatwave_T days. For heatwaves_TH, we found all‐cause ED presentations significantly increased by 6.1% (RR 1.061, 95% CI 1.025–1.098) for severe/extreme heatwave days and remained similar to non‐heatwave days for low‐intensity heatwaves (RR 1.009, 95% CI 0.995–1.024) (Figure 3, Table S2 in Supporting Information S1). When removing air quality from our models in sensitivity analysis (Table S5 in Supporting Information S1), the risk ratio reduced slightly for severe/extreme heatwaves (heatwave_T: RR 1.036, 95% CI 1.023–1.050; heatwave_TH: RR 1.048, 95%CI 1.031–1.065).

#### 3.3.1 Stratification by Demographic Characteristics

Severe or extreme heatwaves significantly increased ED presentations for non‐Aboriginal people by 3.7% (RR 1.037, 95% CI 1.004–1.072) for heatwaves_T and 4.6% (RR 1.046, 95% CI 1.003–1.091) for heatwaves_TH. For Aboriginal peoples, there was a non‐significant increase in ED presentations of 2.2% (RR 1.022, 95% CI 0.987–1.059) during severe/extreme heatwaves_T and a significant increase of 5.9% (RR 1.059, 95% CI 1.006–1.114) for heatwaves_TH (Figure 3, Table S2 in Supporting Information S1).

The risk of people residing in the most disadvantaged socioeconomic indexed regions presenting to ED on severe/extreme heatwave_TH days was almost 1.5 times that of the overall population with an 8.6% increase (RR 1.086, 95% CI 1.019–1.157). In contrast, during severe/extreme heatwaves_T, the 2.4% (RR 1.024, 95% CI 0.98–1.070) increase in ED presentations in the most disadvantaged socioeconomic population was almost half that of the general population.

Among age groups, those aged 19–49 years had increased ED presentations of 5.2% (RR 1.052, 95% CI 1.018–1.087) for severe/extreme heatwaves_T. During severe/extreme heatwaves_TH, risk of ED presentation in ages 50–64 years was over double the general population with an increase of 14.1% (RR 1.141, 95% CI 1.059–1.230).

ED presentations increased 16.2% (RR 1.162, 95% CI 1.038–1.301) among visitors to the NT for severe/extreme heatwaves_T with risk 5 times higher than NT residents (RR 1.031, 95% CI 1.005–1.058).

While increases in ED presentations were observed in both arid and tropical zones, the overall results were not sensitive to climatic differences (Tables S3 and S4 in Supporting Information S1). However, there were differences observed by disease and by demographic characteristics.

#### 3.3.2 Stratification by Presenting Diagnosis

Heat exposure was identified as the condition with the highest risk of ED presentation during severe or extreme heatwaves with an increased risk of 6.717 (95% CI 6.150–7.337) during heatwaves_T and 4.547 (95% CI 4.088–5.058) for heatwaves_TH.

The effect size was sensitive to definition of the heatwaves when examining principal diagnosis (Figure 3, Table S2 in Supporting Information S1). Skin conditions presentations, inclusive of sunburn, abscesses, cellulitis, cysts, dermatitis, and ulcers increased 11.6% (RR 1.116, 95% CI 1.048–1.189) for severe/extreme heatwaves_T. Conversely, presentation for skin conditions did not increase for severe/extreme heatwaves_TH (RR 1.005, 95% CI 0.923–1.094). Digestive conditions, encompassing abnormalities, disease or failures of the gastrointestinal tract, biliary tract, pancreas, liver, or gallbladder increased for severe/extreme heatwaves_T (RR 1.077, 95% CI 1.014–1.144). Cardiovascular conditions such as cardiac arrest, hypertension, arrhythmia, inflammation, congestion or ischemia of the heart were associated with an 11.1% (RR 1.111, 95% CI 1.015–1.216) increase in ED presentations during severe/extreme heatwaves_TH.

## 4 Discussion

We observed a positive dose‐response relationship between heatwave intensity and presentations to EDs in the NT, with all‐cause presentations increasing for heatwaves_T by 4.4% (RR 1.018–1.071) for severe or extreme events and 1.6% (RR 1.016, 95% CI 1.002–1.030) during low‐intensity events. We found the highest effect size for all‐cause ED presentations during severe/extreme heatwaves_TH with a 6.1% increase (RR 1.061, 95% CI 1.025–1.098), while low‐intensity heatwaves_TH showed no significant increase in ED presentations.

Stratifications by subpopulations found unique health impacts for heatwaves_TH, with significant increases in presentations of 5.9% for Aboriginal peoples (RR 1.059, 95% CI 1.006–1.114), 6.3% for males (RR 1.063, 95% CI 1.018–1.110) and 11.1% (RR 1.11, 95%CI 1.015–1.216) for people presenting with cardiovascular conditions. These findings validate inclusion of humidity in heatwave warnings systems, with differing at‐risk profiles highlighting the need to identify vulnerable populations based on heatwave type. This approach informs targeted public health preventative measures and health service preparedness.

Comparable studies from other Australian jurisdictions show NT effect sizes were lower, with interstate studies observing a 4% increase in ED presentations for low‐intensity heatwaves, and 7%–34% for severe, extreme or all‐intensity heatwave days (Campbell et al., 2021; Patel et al., 2019; Scalley et al., 2015; Thomson et al., 2023). The increase in ED presentations in NT is modest when considering daily variations in the number of presentations; however, the cumulative impact of consecutive heatwave days imposes additional strain on already overstretched health services, which, unlike larger cities, have limited options to divert ambulances or patients to nearby hospitals when capacity is exceeded (Jones, 2022). Additionally, ED presentations represent only the tip of the iceberg of true health impacts, as they do not account for the full impact of heat, including heat‐related mortality (estimated at 14% in the NT) (Quilty et al., 2023), ambulance call‐outs (increases in dispatches ranging from 2% to 34%) (Campbell et al., 2021; Mason et al., 2022), and unquantified primary health presentations. While not normally accounted for in mortality and morbidity impact assessments, heat and humidity are also likely to impact workforce productivity and social opportunities in the NT (Campbell et al., 2022).

Neither heatwave index fully identified all heatwaves days. Consequently, relying only on one heatwave metric (dry‐heat or humid heatwaves) would fail to reflect the full population‐level impact. Of the dry‐heat heatwave days, 57.1% were not identified by the heatwave_TH index, while 49.6% of humid heatwave days were not flagged by the heatwave_T index. These findings highlight the importance of incorporating humidity into heatwave monitoring, given its association with health impacts among vulnerable populations in the NT, including older adults, those who are socioeconomically disadvantaged, and people presenting with cardiovascular conditions.

Climate change effects of increasing heatwave_T days were observed in our study from 2001 to 2023. We also noted an increasing number of heatwave_TH days, in both the tropical and arid zones of the NT for low‐intensity heatwaves. This theoretically aligns with the effects of rising greenhouse gas concentrations trapping heat which in turn contributes to warmer air that can retain more water vapor (Baldwin et al., 2023; Jovanovic et al., 2023). We found humidity in arid Australia translated to health effects on the population, leading to our inclusion of the arid zone in our humidity inclusive heatwave analysis. Our findings suggest epidemiological studies should incorporate humidity as an exposure in heat‐related morbidity even when the climate is not tropical. While Baldwin et al. (2023) advocate for alternate methods of incorporating humidity in exposure metrics, we observed impacts on health utilizing the EHF heat index (Nairn et al., 2022).

In contrast to non‐Aboriginal ED presentations (RR 1.037, 95% CI 1.004–1.071) during severe/extreme heatwave_T days, we found that ED presentations among Aboriginal peoples did not quite reach a significant increase compared to non‐heatwave days (RR 1.022, 95% CI 0.987–1.059). Despite this, Aboriginal peoples were highly represented in ED presentations during heatwaves, accounting for 45.3%–49.9% of cases, while comprising only 26% of the population. This elevated baseline aligns with previous publication that shows Aboriginal peoples in the NT have the highest rate of ED presentations in Australia (1,262.2 per 1,000 population), which is 2.2 times higher than that of non‐Aboriginal people in the NT (Australian Institute of Health and Welfare, 2025). Conversely, during severe/extreme heatwaves_TH, Aboriginal and non‐Aboriginal ED presentations significantly increased by 5.9% (RR 1.059, 95% CI 1.006–1.114) and 4.6% (RR 1.046, 95% CI 1.003–1.091) respectively. These findings are consistent with Quilty et al. (2023) who found similar mortality among NT Aboriginal and non‐Aboriginal peoples during extreme temperature, despite significant socioeconomic disparities. Quilty et al. (2023) suggests Aboriginal peoples' social and cultural adaptations to extreme environments, developed over millennia, offer valuable insights for adapting to excess heat. Australia's National Health and Climate Strategy highlights the importance of embracing First Nations knowledge as an opportunity to collectively rethink our relationship with how we live and work in an increasingly hostile environment to strengthen resilience (Australian Government, 2023).

Variation in effect size by EHF definition were found by age groups. Working age people of 19–49 years were most affected by severe/extreme heatwaves_T, with a 5.2% (RR 1.052, 95% CI 1.018–1.087) increase in ED presentations. While 19–49 year olds had a 4.1% increase (RR 1.041, 95%CI 0.997–1.087) during severe/extreme heatwave_TH presentations, this increase did not reach significance. In contrast, older people aged 50–64 years were most affected by heatwave_TH with a 14.1% (RR 1.141, 95% CI 1.059–1.230) increase during severe/extreme events, an effect not so pronounced during heatwaves_T (RR 1.028, 95% CI 0.975–1.085). While the risk factors of older age, existing chronic conditions and performing physical work are known risk factors for insufficient thermoregulation during extreme heat (Kenny et al., 2010), the differing impacts across age groups that appear dependent on whether humidity is directly included in the heatwave index requires further investigation. It is likely that the small sample size of individuals over 64 years limited the statistical precision needed to detect an effect in this known vulnerable population. Previous research suggests that older adults have diminished thermoregulatory responses to heat stress, regardless of whether conditions are dry or humid (McKenna et al., 2023). Additionally, cooling strategies such as electric fan use may not prevent heat‐related physiological stress for older adults (Gagnon et al., 2016).

Heat exposure showed the most significant increase in presentations, consistent with other study (Mason et al., 2022), but this accounted for only a minority of presentations. Strain on other body systems, particularly exacerbated by chronic conditions, results from physiological adaptations to heat, such as sweating and blood flow redistribution for thermoregulation (Ebi et al., 2021). Hence, similar to previous studies (Mason et al., 2022), we identified increases in a range of presenting diagnoses, including patient presentations with diabetes (heatwaves_T), skin conditions noting inclusion of sunburn among conditions (heatwaves_T), digestive (heatwaves_T and heatwave_TH) and cardiovascular conditions (heatwave_TH). While it is noteworthy that diagnostic presentations were more prevalent in accordance with the heatwave index measure applied, interpretation of these results are challenging due to small numbers affecting precision of estimates. Regardless, these findings reinforce the importance of including both heatwave indexes of heatwaves_T and heatwaves_TH to capture the true impact of dry and humid heatwave events on health.

We studied visitor presentations following ED physician feedback noting disproportionate representation during heatwaves, and confirmed visitor presentations increased by 16.2% (RR 1.162, 95% CI 1.038–1.301) during heatwaves_T. This was higher than NT residents who showed a 3.1% (RR 3.1, 95% CI 1.005–1.058) increase for these days. While visitors should be targeted for warnings and strategies to prevent heat stress during severe and extreme heatwaves, whole of population public health messaging is also important. It is crucial for Territorians to move beyond the attitude of “we're Territory tough” (Campbell et al., 2022) and implement proactive heatwave response plans when heatwave warnings are issued.

### 4.1 Policy Implications

Our study highlights the tangible impacts of dry‐heat and humid‐heat heatwaves on the health of Territorians and importance of hazard plans and ongoing surveillance in tropical and arid regions such as the NT.

Greater recognition of the combined health impacts of heat and humidity is needed. Consideration should be given to prioritizing the development and integration of a direct humidity metric to strengthen Australia's early warning system for identifying and responding to increasingly frequent climate‐related health events. As neither heatwave index fully captured all heatwave days and both indexes were independently associated with unique health outcomes, the NT would benefit from a heatwave warning system based on both EHF temperature‐only (heatwave_T) and EHF heat index (heatwave_TH). While this approach could improve identification of risk, we acknowledge the challenges of forecasting humidity (Nairn et al., 2022), and the potential for warning fatigue with more frequent community‐wide heatwave alerts. To maintain effectiveness, public health responses should focus on high‐impact, severe/extreme events, with targeted strategies for vulnerable populations, such as older adults, socioeconomically disadvantaged groups, and people with cardiovascular conditions. Rather than relying solely on broad public alerts, response planning should focus on local service providers and community organizations better placed to reach and support those most at risk.

The NT health workforce itself must be protected, as evidence shows that extreme heat exposure contributes to stress and staff attrition in already overburdened systems (Pendrey et al., 2021). Ensuring the wellbeing of the NT health workforce, alongside the capacity of health services to safely respond to forecast surges in demand, is essential to building a robust and climate‐resilient health system.

### 4.2 Strengths and Limitations

Our study is unique in examining the health effects of humidity in combination with temperature by adding humidity to the excess heat factor index. This approach aligns with recommendations from meteorological experts who have highlighted the inadequacy of Australia's temperature‐based heatwave warning system for tropical regions (Nairn et al., 2022). A further strength of our study is the inclusion of all NT EDs, enabled by the NT Department of Health's exclusive provision of ED services and facilitated through the use of one record management system across all hospitals. We undertook an in‐depth analysis across various population subgroups and heatwave definitions, which allowed us to identify the most vulnerable populations in a region with some existing adaptation to already extreme heat conditions. We controlled air quality in our main models, acknowledging debate of the role of air quality role as a mediator or confounder of heat‐health relationships (Buckley et al., 2014). During severe/extreme heatwaves, the whole‐of‐population estimates showed minimal sensitivity to the inclusion or exclusion of PM_2.5_. This was reflected in the overlapping confidence intervals and the slightly lower RR when PM_2.5_ was removed, suggesting that PM_2.5_ is unlikely to function as a mediator in this setting. A limitation of the study was the assumption that individual's heatwave exposure occurred within the SA2 of the residential address or hospital of attendance for visitors, which may have misrepresented the actual location of heat exposure. The ED shortlist of ICD‐10 codes limited the specificity of diagnoses, with heatwave related codes such as X30, 'exposure to excessive natural heat’ absent from the shortlisted codes. Further, our study did not account for delayed presentations in the days following heatwaves, thus may not have identified lagged presentations which are more common for certain conditions such as mental health.

### 4.3 Future Research

This is the first heatwave and health impact study we know of in Australia to compare temperature only index and temperature‐plus‐humidity index utilizing the EHF. Our findings suggest that other regions in Australia include humidity as an exposure when conducting heatwave and health impact studies. There is also a need to assess the economic burden of heatwaves in the NT, to help policymakers allocate adequate funding to health promotion and heat illness prevention. Further, more in‐depth research is needed to understand the social determinants of heatwave illness and reduce barriers to following public health guidelines to keep cool and hydrated. Understanding the home and community environment to which patients return once discharged is critical to understanding if there are preventable factors at play, and what policy levers can be implemented to reduce risks.

## 5 Conclusion

Despite the regularity of high temperatures throughout the NT and acclimatization of Territorians, our study findings demonstrate that heatwaves significantly impact health outcomes, placing additional demand on health services. We observed a dose‐response relationship, with ED presentations increasing as heatwave intensity rose, indicating more people reaching the physiological limits of human adaptive capacity during severe and extreme heatwaves. Considering this relationship, health promotion efforts should prioritize severe and extreme heatwaves while maintaining broader heat‐related strategies. We also found humidity in combination with excess temperature has health impacts in both tropical and arid regions. Each index was independently associated with an increase in ED presentations with variations of sub‐groups impacted, indicating different subpopulation vulnerabilities. Humidity therefore warrants further epidemiological study in heatwave and human health impact studies across a variety of climates. The NT heatwave warning system should consider incorporating the EHF heat index (humidity inclusive index) alongside the current BoM EHF temperature index to support public health interventions aimed at minimizing health impacts for the NT population and visitors.

## Conflict of Interest

The authors declare no conflicts of interest relevant to this study.

## Supporting information

Supporting Information S1

## Data Availability

Data supporting this research are available by request from NT Health (email datareleaserequests.doh@nt.gov.au or visit https://health.nt.gov.au/data‐and‐research/health‐data/how‐to‐request‐data‐for‐secondary‐use); Bureau of Meteorology (email climatedata@bom.gov.au or visit https://reg.bom.gov.au/climate/data‐services/station‐data.shtml); and Clean Air Research Data and Analysis Tools (CARDAT) (email car.data@curtin.edu.au or visit https://cardat.github.io/data_access_request_form.html).
