# Supplementary material for: Emergency Department Presentations During Dry and Humid Heatwaves: A Case‐Crossover Study in the Northern Territory, Australia
Source: Geohealth. 2026 May 29;10(6):e2025GH001562. doi: 10.1029/2025GH001562 (PMC13238576; doi:10.1029/2025GH001562)
Supplement: Supplementary file 1 — Supporting Information S1 [file GH2-10-e2025GH001562-s001.pdf]

GeoHealth

Supporting Information for

Emergency Department Presentations During Dry and Humid Heatwaves: A Case-Crossover Study in the Northern Territory, Australia

Rowena Boyd[1], Alyson Wright[1], Nicolas Borchers-Arriagada[2], Paul Fox-Hughes[3], Fay H. Johnston[2,4], Paul Burgess[1], Tracy Ward[1], Sharon L. Campbell[2]

[1]Public Health Directorate, Department of Health, Northern Territory, Australia
[2]Menzies Institute for Medical Research, University of Tasmania, Australia
[3]Bureau of Meteorology, Hobart, Tasmania, Australia
[4]Public Health Services, Department of Health, Tasmania, Australia

Contents of this file

Introduction
Supplementary Tables S1 to S5

Introduction

Supplementary Tables S1-S5 contain additional data as outlined in each table caption.

Table S1. Percentage of days meeting heatwave conditions and percentage point difference by climate zone, heatwave index and intensity, Northern Territory, 2001-2005 and 2019-2023.

Heatwave index	Climatic zone	Heatwave intensity	Percentage of days meeting heatwave conditions during 5-year period		Percentage point difference
			2001-2005	2019-2023	
Temperature only	Tropical	Low	4.2%	7.1%	2.9
Temperature only	Tropical	Severe/extreme	0.9%	1.5%	0.6
Temperature only	Arid	Low	2.1%	5.3%	3.2
Temperature only	Arid	Severe/extreme	0.4%	1.4%	1.0
Temperature + humidity	Tropical	Low	3.2%	4.9%	1.7
Temperature + humidity	Tropical	Severe/extreme	0.5%	1.0%	0.5
Temperature + humidity	Arid	Low	2.5%	5.0%	2.5
Temperature + humidity	Arid	Severe/extreme	0.7%	0.5%	-0.2

Table S2. Association between Northern Territory emergency department presentations and heatwaves (temperature-only and temperature-plus-humidity), by characteristic, presenting diagnosis and heatwave intensity, 2001-2023.

	Heatwave - Temperature only		Heatwave - Temperature + humidity	
	Low intensity	Severe/extreme intensity	Low intensity	Severe/extreme intensity
**Whole NT**	Risk Ratio (95% confidence interval)			
**Whole population**	1.016 (1.002 - 1.030)*	1.044 (1.018 - 1.071)*	1.009 (0.995 - 1.024)	1.061 (1.025 - 1.098)*
**Indigenous status**				
Aboriginal	1.006 (0.987 - 1.025)	1.022 (0.987 - 1.059)	0.998 (0.979 - 1.018)	1.059 (1.006 - 1.114)*
Non-Aboriginal	1.008 (0.990 - 1.026)	1.037 (1.004 - 1.072)*	1.004 (0.985 - 1.023)	1.046 (1.003 - 1.091)*
**Sex**				
Male	1.008 (0.990 - 1.026)	1.022 (0.990 - 1.055)	0.999 (0.981 - 1.018)	1.063 (1.018 - 1.110)*
Female	1.016 (0.998 - 1.034)	1.059 (1.025 - 1.094)*	1.004 (0.985 - 1.023)	1.060 (1.014 - 1.108)*
**Index of Relative Socio-economic Disadvantage (IRSD decile)**				
Disadvantaged (0-3)	0.999 (0.976 - 1.023)	1.024 (0.980 - 1.070)	0.984 (0.960 - 1.008)	1.086 (1.019 - 1.157)
Moderate (4-6)	1.022 (1.000 - 1.045)*	1.038 (0.998 - 1.079)	1.003 (0.980 - 1.026)	1.037 (0.981 - 1.097)
Advantaged (7-10)	1.020 (0.994 - 1.047)	1.049 (0.999 - 1.101)	1.037 (1.009 - 1.066)*	1.051 (0.995 - 1.111)
**Age group**				
Age < 5 years	1.025 (0.988 - 1.063)	0.977 (0.914 - 1.043)	1.013 (0.974 - 1.052)	1.009 (0.919 - 1.107)
5 to 18 years	0.996 (0.965 - 1.029)	0.990 (0.935 - 1.049)	0.990 (0.957 - 1.025)	0.972 (0.898 - 1.052)
19 to 49 years	1.011 (0.993 - 1.028)	1.052 (1.018 - 1.087)*	1.003 (0.985 - 1.022)	1.041 (0.997 - 1.087)
50 to 64 years	0.996 (0.968 - 1.026)	1.028 (0.975 - 1.085)	1.036 (1.004 - 1.069)*	1.141 (1.059 - 1.230)*
> 64 years	1.005 (0.964 - 1.049)	1.016 (0.940 - 1.097)	1.026 (0.981 - 1.074)	1.054 (0.952 - 1.166)
**Residential status**				
NT resident	1.016 (1.002 - 1.030)*	1.031 (1.005 - 1.058)*	1.004 (0.989 - 1.019)	1.053 (1.017 - 1.090)*
Visitor	0.972 (0.913 - 1.035)	1.162 (1.038 - 1.301)*	1.028 (0.963 - 1.097)	1.111 (0.957 - 1.290)
**Principal diagnostic condition**				
Effects Heat/light	1.734 (1.670 - 1.800)*	6.717 (6.150 - 7.337)*	1.724 (1.663 - 1.787)*	4.547 (4.088 - 5.058)*
Diabetes	1.071 (1.022 - 1.123)*	0.891 (0.832 - 0.954)	0.720 (0.687 - 0.755)	1.027 (0.917 - 1.151)
Skin	1.004 (0.970 - 1.038)	1.116 (1.048 - 1.189)*	0.990 (0.956 - 1.025)	1.005 (0.923 - 1.094)
Respiratory	1.027 (0.994 - 1.061)	1.013 (0.954 - 1.076)	0.982 (0.949 - 1.017)	1.029 (0.947 - 1.118)
Infectious	0.998 (0.962 - 1.035)	1.031 (0.965 - 1.101)	0.990 (0.952 - 1.030)	1.011 (0.920 - 1.111)
Cardiovascular	0.995 (0.959 - 1.032)	0.988 (0.924 - 1.056)	0.976 (0.939 - 1.014)	1.111 (1.015 - 1.216)*
Musculoskeletal	1.008 (0.973 - 1.044)	1.011 (0.950 - 1.077)	0.995 (0.959 - 1.033)	0.950 (0.871 - 1.037)
Urinary	0.972 (0.934 - 1.012)	0.969 (0.899 - 1.045)	0.943 (0.904 - 0.984)	1.012 (0.917 - 1.118)
Injury	0.999 (0.973 - 1.026)	1.003 (0.955 - 1.053)	0.967 (0.940 - 0.994)	0.977 (0.915 - 1.043)
Digestive	1.016 (0.982 - 1.050)	1.077 (1.014 - 1.144)*	1.038 (1.002 - 1.076)*	0.929 (0.854 - 1.010)
Mental/behavioural	0.979 (0.944 - 1.016)	0.926 (0.865 - 0.991)	1.010 (0.972 - 1.048)	0.943 (0.860 - 1.035)

*Statistically significant higher emergency department presentations

Table S3. Association between NT emergency department presentations and heatwaves (temperature-only and temperature-plus-humidity), by characteristic, presenting diagnosis and heatwave intensity, tropical climate, 2001-2023.

Tropical climate	Heatwave - Temperature only		Heatwave - Temperature + humidity	
	Low intensity	Severe/extreme intensity	Low intensity	Severe/extreme intensity
	Risk Ratio (95% confidence interval)			
**Whole population**	1.020 (1.003 - 1.036)	1.038 (1.009 - 1.068)	1.015 (0.997 - 1.033)	1.053 (1.013 - 1.094)
**Indigenous status**				
Aboriginal	1.004 (0.981 - 1.028)	0.977 (0.939 - 1.017)	0.988 (0.963 - 1.013)	1.056 (0.994 - 1.122)
Non-Aboriginal	1.009 (0.989 - 1.029)	1.047 (1.012 - 1.084)	1.012 (0.991 - 1.033)	1.032 (0.987 - 1.078)
**Sex**				
Male	1.008 (0.989 - 1.029)	1.018 (0.983 - 1.054)	1.001 (0.980 - 1.023)	1.050 (1.002 - 1.101)
Female	1.020 (0.999 - 1.041)	1.046 (1.009 - 1.084)	1.009 (0.987 - 1.032)	1.059 (1.008 - 1.112)
**Index of Relative Socio-economic Disadvantage (IRSD decile)**				
Disadvantaged (0-3)	1.009 (0.979 - 1.040)	0.987 (0.934 - 1.044)	0.976 (0.944 - 1.009)	1.077 (0.993 - 1.168)
Moderate (4-6)	1.015 (0.990 - 1.042)	1.031 (0.989 - 1.076)	1.000 (0.973 - 1.028)	1.033 (0.968 - 1.102)
Advantaged (7-10)	1.024 (0.997 - 1.051)	1.055 (1.004 - 1.108)	1.038 (1.009 - 1.068)	1.050 (0.993 - 1.110)
**Age group**				
Age < 5 years	1.037 (0.995 - 1.081)	0.961 (0.894 - 1.032)	1.016 (0.971 - 1.063)	0.976 (0.878 - 1.086)
5 to 18 years	1.004 (0.968 - 1.040)	0.967 (0.909 - 1.028)	0.998 (0.959 - 1.039)	0.954 (0.875 - 1.039)
19 to 49 years	1.011 (0.991 - 1.032)	1.057 (1.020 - 1.095)	1.015 (0.994 - 1.038)	1.049 (0.999 - 1.100)
50 to 64 years	0.987 (0.954 - 1.021)	1.043 (0.985 - 1.105)	1.053 (1.015 - 1.092)	1.086 (1.000 - 1.179)
> 64 years	1.017 (0.969 - 1.066)	0.978 (0.900 - 1.063)	1.012 (0.962 - 1.065)	0.983 (0.881 - 1.097)
**Residential status**				
NT resident	1.018 (1.002 - 1.035)	1.023 (0.995 - 1.053)	1.006 (0.989 - 1.024)	1.047 (1.007 - 1.088)
Visitor	0.984 (0.903 - 1.073)	1.176 (1.025 - 1.350)	1.059 (0.967 - 1.158)	1.116 (0.922 - 1.351)
**Principal diagnostic condition**				
Effects Heat/light	1.253 (1.201 - 1.306)	5.062 (4.646 - 5.516)	1.736 (1.668 - 1.808)	4.681 (4.224 - 5.188)
Diabetes	0.798 (0.754 - 0.845)	0.885 (0.831 - 0.942)	0.688 (0.653 - 0.725)	0.779 (0.689 - 0.882)
Skin	1.022 (0.984 - 1.061)	1.092 (1.021 - 1.168)	0.970 (0.932 - 1.010)	1.001 (0.914 - 1.097)
Respiratory	1.020 (0.984 - 1.057)	1.002 (0.940 - 1.068)	0.979 (0.942 - 1.018)	1.002 (0.917 - 1.096)
Infectious	0.982 (0.944 - 1.023)	1.001 (0.934 - 1.074)	0.974 (0.932 - 1.017)	0.949 (0.860 - 1.047)
Cardiovascular	1.000 (0.961 - 1.040)	1.050 (0.979 - 1.126)	0.939 (0.900 - 0.980)	1.029 (0.936 - 1.131)
Musculoskeletal	1.012 (0.973 - 1.052)	1.027 (0.961 - 1.098)	1.017 (0.976 - 1.061)	0.910 (0.830 - 0.999)
Urinary	1.063 (1.017 - 1.111)	1.002 (0.926 - 1.083)	0.946 (0.903 - 0.992)	1.050 (0.947 - 1.165)
Injury	1.001 (0.971 - 1.031)	0.993 (0.942 - 1.046)	0.957 (0.926 - 0.989)	0.972 (0.904 - 1.045)
Digestive	1.013 (0.976 - 1.052)	1.082 (1.015 - 1.154)	1.025 (0.985 - 1.068)	0.928 (0.849 - 1.015)
Mental/behavioural	1.016 (0.976 - 1.059)	0.878 (0.818 - 0.942)	0.966 (0.925 - 1.008)	0.931 (0.843 - 1.028)

*Statistically significant higher emergency department presentations

Table S4. Association between NT emergency department presentations and heatwaves (temperature-only and temperature-plus-humidity), by characteristic, presenting diagnosis and heatwave intensity, arid climate, 2001-2023.

	Heatwave - Temperature only		Heatwave - Temperature + humidity	
	Low intensity	Severe/extreme intensity	Low intensity	Severe/extreme intensity
**Arid climate**	Risk Ratio (95% confidence interval)			
**Whole population**	1.006 (0.979 - 1.034)	1.066 (1.005 - 1.129)*	0.998 (0.971 - 1.026)	1.087 (1.007 - 1.173)*
**Indigenous status**				
Aboriginal	1.006 (0.973 - 1.040)	1.108 (1.035 - 1.186)*	1.008 (0.976 - 1.042)	1.061 (0.969 - 1.162)
Non-Aboriginal	1.008 (0.964 - 1.053)	0.981 (0.892 - 1.080)	0.978 (0.935 - 1.024)	1.131 (1.001 - 1.278)*
**Sex**				
Male	1.007 (0.970 - 1.045)	1.034 (0.958 - 1.116)	0.996 (0.960 - 1.033)	1.108 (1.000 - 1.227)*
Female	1.008 (0.973 - 1.043)	1.101 (1.022 - 1.186)*	0.995 (0.960 - 1.030)	1.064 (0.967 - 1.171)
**Index of Relative Socio-economic Disadvantage (IRSD decile)**				
Disadvantaged (0-3)	0.986 (0.950 - 1.024)	1.079 (1.003 - 1.161)	0.991 (0.955 - 1.028)	1.097 (0.990 - 1.216)
Moderate (4-6)	1.037 (0.995 - 1.081)	1.060 (0.963 - 1.168)	1.009 (0.967 - 1.053)	1.049 (0.937 - 1.175)
Advantaged (7-10)	0.977 (0.875 - 1.091)	0.923 (0.705 - 1.209)	1.027 (0.915 - 1.152)	1.108 (0.803 - 1.529)
**Age group**				
Age < 5 years	0.999 (0.932 - 1.070)	1.032 (0.897 - 1.188)	1.009 (0.942 - 1.080)	1.077 (0.902 - 1.285)
5 to 18 years	0.978 (0.917 - 1.043)	1.076 (0.943 - 1.228)	0.979 (0.919 - 1.044)	1.025 (0.866 - 1.213)
19 to 49 years	1.009 (0.975 - 1.045)	1.039 (0.965 - 1.118)	0.981 (0.948 - 1.016)	1.021 (0.927 - 1.124)
50 to 64 years	1.014 (0.960 - 1.071)	0.984 (0.875 - 1.106)	1.012 (0.956 - 1.071)	1.289 (1.101 - 1.509)*
> 64 years	0.978 (0.899 - 1.064)	1.167 (0.973 - 1.399)	1.071 (0.977 - 1.175)	1.357 (1.074 - 1.715)*
**Residential status**				
NT resident	1.009 (0.981 - 1.038)	1.061 (0.999 - 1.126)	0.999 (0.971 - 1.028)	1.076 (0.995 - 1.165)
Visitor	0.967 (0.884 - 1.059)	1.132 (0.930 - 1.377)	1.006 (0.917 - 1.105)	1.095 (0.863 - 1.390)
**Principal diagnostic condition**				
Effects Heat/light	Small numbers	Small numbers	Small numbers	Small numbers
Diabetes	1.398 (1.259 - 1.553)*	0.683 (0.530 - 0.881)	0.769 (0.688 - 0.859)	1.568 (1.177 - 2.089)*
Skin	0.966 (0.892 - 1.046)	1.211 (1.023 - 1.433)*	1.024 (0.948 - 1.105)	1.012 (0.816 - 1.255)
Respiratory	1.043 (0.964 - 1.128)	1.042 (0.890 - 1.219)	0.989 (0.913 - 1.071)	1.101 (0.893 - 1.358)
Infectious	1.032 (0.945 - 1.126)	1.120 (0.940 - 1.335)	1.028 (0.938 - 1.127)	1.242 (0.959 - 1.609)
Cardiovascular	0.983 (0.898 - 1.075)	0.732 (0.600 - 0.894)	1.101 (1.004 - 1.208)*	1.548 (1.198 - 1.999)*
Musculoskeletal	0.998 (0.918 - 1.084)	0.953 (0.804 - 1.129)	0.951 (0.874 - 1.035)	1.089 (0.869 - 1.365)
Urinary	0.813 (0.738 - 0.896)	0.905 (0.735 - 1.113)	0.937 (0.849 - 1.035)	0.919 (0.704 - 1.201)
Injury	0.993 (0.937 - 1.053)	1.045 (0.922 - 1.185)	0.989 (0.933 - 1.048)	0.995 (0.850 - 1.166)
Digestive	1.022 (0.947 - 1.104)	1.056 (0.898 - 1.241)	1.068 (0.986 - 1.157)	0.933 (0.745 - 1.169)
Mental/behavioural	0.913 (0.840 - 0.994)	1.119 (0.932 - 1.344)	1.080 (0.993 - 1.174)	0.969 (0.763 - 1.231)

*Statistically significant higher emergency department presentations

Table S5. Association between Northern Territory emergency department presentations and heatwaves (temperature-only and temperature-plus-humidity), by characteristic, presenting diagnosis and heatwave intensity, 2001-2023 for all Northern Territory, with air quality removed as a covariate.

	Heatwave - Temperature only		Heatwave - Temperature + humidity	
	Low intensity	Severe/extreme intensity	Low intensity	Severe/extreme intensity
**Whole NT**	Risk Ratio (95% confidence interval)			
**Whole population**	1.016 (1.010 - 1.023)*	1.036 (1.023 - 1.050)*	1.015 (1.008 - 1.022)*	1.048 (1.031 - 1.065)*
**Indigenous status**				
Aboriginal	1.008 (1.000 - 1.017)*	1.024 (1.007 - 1.041)*	1.011 (1.002 - 1.020)*	1.047 (1.024 - 1.069)*
Non-Aboriginal	1.018 (1.010 - 1.027)*	1.032 (1.015 - 1.049)*	1.014 (1.005 - 1.023)*	1.039 (1.018 - 1.060)*
**Sex**				
Male	1.011 (1.003 - 1.019)*	1.019 (1.003 - 1.036)*	1.011 (1.002 - 1.020)*	1.043 (1.022 - 1.064)*
Female	1.015 (1.006 - 1.023)*	1.040 (1.023 - 1.056)*	1.009 (1.000 - 1.017)*	1.035 (1.014 - 1.056)*
**Index of Relative Socio-economic Disadvantage (IRSD decile)**				
Disadvantaged (0-3)	1.012 (1.001 - 1.023)*	1.020 (0.998 - 1.041)	1.015 (1.000 - 1.031)*	1.007 (0.970 - 1.045)
Moderate (4-6)	1.022 (1.012 - 1.033)*	1.030 (1.010 - 1.051)*	1.013 (1.002 - 1.024)*	1.044 (1.017 - 1.071)*
Advantaged (7-10)	1.014 (1.001 - 1.026)*	1.046 (1.021 - 1.072)*	1.012 (0.999 - 1.026)	1.050 (1.022 - 1.079)*
**Age group**				
Age < 5 years	1.017 (1.003 - 1.033)*	1.007 (0.978 - 1.037)	1.015 (1.000 - 1.031)*	1.007 (0.970 - 1.045)
5 to 18 years	1.007 (0.993 - 1.021)	1.012 (0.985 - 1.039)	1.006 (0.991 - 1.021)	0.993 (0.961 - 1.027)
19 to 49 years	1.016 (1.008 - 1.024)*	1.027 (1.011 - 1.044)*	1.013 (1.005 - 1.022)*	1.028 (0.974 - 1.085)
50 to 64 years	0.995 (0.983 - 1.007)	1.018 (0.994 - 1.042)	0.997 (0.984 - 1.010)	1.066 (1.034 - 1.098)*
> 64 years	1.000 (0.983 - 1.017)	1.004 (0.973 - 1.036)	1.009 (0.991 - 1.028)	1.025 (0.984 - 1.067)
**Residential status**				
NT resident	1.018 (1.011 - 1.024)*	1.031 (1.017 - 1.044)*	1.013 (1.006 - 1.020)*	1.043 (1.026 - 1.060)*
Visitor	0.988 (0.959 - 1.017)	1.065 (1.006 - 1.128)*	1.007 (1.004 - 1.052)*	1.038 (0.966 - 1.116)
**Principal diagnostic condition**				
Effects Heat/light	1.941 (1.896 - 1.988)*	1.920 (1.850 - 1.993)*	1.013 (0.993 - 1.034)	1.334 (1.262 - 1.411)*
Diabetes	1.071 (1.038 - 1.105)*	1.201 (1.136 - 1.270)*	0.949 (0.919 - 0.980)	1.124 (1.042 - 1.213)*
Skin	1.010 (0.990 - 1.030)	1.050 (1.011 - 1.091)*	1.013 (0.993 - 1.034)	1.005 (0.959 - 1.053)
Respiratory	1.034 (1.016 - 1.053)*	1.021 (0.985 - 1.057)	0.997 (0.978 - 1.016)	0.989 (0.945 - 1.034)
Infectious	0.994 (0.973 - 1.016)	1.036 (0.994 - 1.081)	1.028 (1.004 - 1.052)*	1.028 (0.974 - 1.085)
Cardiovascular	0.994 (0.972 - 1.015)	1.031 (0.989 - 1.075)	1.010 (0.987 - 1.033)	1.025 (0.973 - 1.080)
Musculoskeletal	0.992 (0.972 - 1.013)	1.042 (1.002 - 1.083)*	0.999 (0.978 - 1.020)	1.023 (0.973 - 1.076)
Urinary	1.002 (0.977 - 1.028)	1.017 (0.968 - 1.068)	0.998 (0.972 - 1.025)	1.091 (1.027 - 1.160)*
Injury	1.007 (0.993 - 1.021)	1.019 (0.993 - 1.046)	1.004 (0.990 - 1.018)	1.018 (0.985 - 1.052)
Digestive	0.998 (0.980 - 1.017)	1.017 (0.982 - 1.054)	1.017 (0.997 - 1.037)	0.990 (0.946 - 1.036)
Mental/behavioural	0.982 (0.961 - 1.003)	0.950 (0.911 - 0.990)	1.008 (0.986 - 1.031)	0.952 (0.902 - 1.004)

*Statistically significant higher emergency department presentations
